# Focus Issue on Squamous Cell Carcinoma: Practical Concerns Regarding the 7th Edition AJCC Staging Guidelines

**DOI:** 10.1155/2011/156391

**Published:** 2010-11-22

**Authors:** D. Buethe, C. Warner, J. Miedler, C. J. Cockerell

**Affiliations:** ^1^Department of Dermatology, University of Texas Southwestern Medical Center, 5323 Harry Hines Blvd, Dallas, TX 75390-9191, USA; ^2^Cockerell and Associates Dermatopathology Laboratories/Dermpath Diagnostics, 2330 Butler St., Suite 115, Dallas, TX 75235-7800, USA

## Abstract

The 7th edition of the AJCC Cancer Staging Manual represents a dramatic shift in the way that cutaneous squamous cell carcinoma (cSCC) is staged, in that it is first attempt to incorporate evidence-based medicine into the staging guidelines for cSCC. In our opinion, the changes made to the seventh edition represent a significant improvement over previous editions and will ultimately lead to improved patient stratification, more accurate prognostic data, and a better framework to guide clinical decision making. However, there are a number of issues within the latest guidelines that require clarification or are impractical for clinical practice. The purpose of this paper is to highlight the key changes to the 6th edition staging manual as they pertain to cSCC, to point out impractical component of the 7th edition and/or aspects that require further clarification, and to make recommendations that address any current shortcomings to improve subsequent editions. Specific focus will be given to the inclusion of separate guidelines for cSCC and Merkel cell carcinoma (MCC), the incorporation of high-risk factors as modifiers of T stage, the addition of new guidelines for advanced T stage, and the changes in stratification of lymph node status. This paper is modified from a more comprehensive treatment of the staging of nonmelanoma skin cancer by Warner and Cockerell entitled *“The new 7th edition American joint committee on cancer staging of cutaneous nonmelanoma skin cancer: a critical review,”* in the American Journal of Clinical Dermatology (paper accepted, pending publication).

## 1. Introduction

The seventh edition of the AJCC Cancer Staging Manual represents a dramatic shift in the way that cutaneous squamous cell carcinoma (cSCC) is staged, in that it is the first attempt to incorporate evidence-based medicine into the staging guidelines for cSCC. In our opinion, although the changes represent a significant improvement over previous editions and will likely lead to improved patient stratification, more accurate prognostic data and a better framework to guide clinical decision making, there are a number of issues that require clarification and that are impractical for clinical practice. The purpose of this paper is to highlight the key changes to the 6th edition staging manual as they pertain to cSCC, to point out impractical components of the 7th edition and/or aspects that require further clarification, and to make recommendations that address any current shortcomings to improve subsequent editions. Specific focus will be given to the inclusion of separate guidelines for cSCC and Merkel cell carcinoma (MCC), the incorporation of high-risk factors as modifiers of T stage, the addition of new guidelines for advanced T stage, and the changes in stratification of lymph node status [1]. This paper is modified from a more comprehensive treatment of the staging of nonmelanoma skin cancer by Warner and Cockerell entitled *“The new 7th edition american joint committee on cancer staging of cutaneous nonmelanoma skin cancer: a critical review,”* in the American Journal of Clinical Dermatology (paper accepted, pending publication) [2]. 

*New Changes to 6th Edition (see Table 1)*.

## 2. Creation of Separate Guidelines for Cutaneous Squamous Cell Carcinoma and Merkel Cell Carcinoma

One of the most dramatic changes to the AJCC 6th edition is the creation of separate staging guidelines for cSCC and Merkel cell carcinoma. In previous versions, these two cancers were grouped together, along with 80 other cutaneous neoplasms, under a single chapter entitled “Carcinoma of the Skin” [3, 4]. This chapter has been eliminated with the 7th edition and replaced by 2 separate chapters: “Merkel Cell Carcinoma” and “Cutaneous Squamous Cell Carcinoma and Other Cutaneous Skin Cancers.” Under this format, all Nonmelanoma, non-Merkel cell skin cancers are to be staged under the cSCC staging system [1].

The creation of separate guidelines represents a significant improvement over previous editions, as cSCC and Merkel cell carcinoma exhibit markedly different behavior. In general, cSCC portends a much more favorable prognosis, with a cure rate greater than 90%, and a disease-specific yearly mortality rate of approximately 1% per year in the U.S [5, 6]. Additionally, the five year recurrence and metastatic rates for cSCC are 8% and 5%, respectively [5, 7, 8]. In contrast, MCC tends to be much more aggressive, with an overall disease-specific survival rate of 76% and overall recurrence and metastatic rates of 52% and 31%, respectively [9–11]. Compared to cSCC, MCC tends to exhibit lymphatic spread earlier in the disease course, as up to 14% of patients present with palpable lymph nodes in the absence of an identifiable primary tumor [12], and nearly a third of patients without clinically evident lymph nodes have evidence of lymphatic spread on sentinel lymph node biopsy [13]. Similarly, while histologic grade, depth of invasion, and perineural involvement have been shown to have important prognostic features for cSCC [5, 14], a significant association has not been demonstrated for MCC [15, 16].

Given the significant differences in clinical behavior, application of a single set of guidelines to both cSCC and MCC can be problematic. Guidelines tailored towards MCC may lead to upstaging of cSCC lesions, placing an unnecessary emotional burden on patients and exposing them to significantly morbid treatments they may not require. Conversely, guidelines designed for cSCC may understage MCC patients, preventing them from receiving the more aggressive therapy they require. In particular, there is some evidence that understaging of MCC lesions may have been a problem with the 6th edition guidelines [16]. 

However, this shortcoming is largely rectified in the 7th edition AJCC staging manual through the creation of separate staging guidelines oriented towards cSCC and MCC, respectively. The chapter entitled “Merkel Cell Carcinoma” is specific to MCC, leading to greater applicability and accuracy of guidelines for patients with this disease. As its name implies, the chapter entitled “Cutaneous Squamous Cell Carcinoma and other Cutaneous Skin Cancers” is primarily oriented towards cSCC. These changes highlight a shift towards the creation of more cancer specific staging guidelines, improving their ability to assess prognosis and guide treatment decisions.

While these changes represent a significant improvement over previous editions, they also highlight an important issue that still remains. Despite a greater focus on cSCC, the AJCC staging manual still groups all Nonmelanoma, non-Merkel cell skin cancers, a diverse set of over 80 different types of tumors, under a single set of staging guidelines [1, 3]. As a result, the applicability of these guidelines to any individual cancer is impaired. Pertaining to cSCC, there is significant variation regarding clinical behavior amongst different variants/subtypes. This leads to several instances where the application of the guidelines may be inappropriate, particularly for less aggressive variants. Although locally aggressive, the verrucous form of cSCC tends to exhibit a less aggressive clinical course than other subtypes and rarely metastasizes [2]. Similarly, keratoacanthomas exhibit predominantly benign clinical behavior, and usually only require conservative treatment for cure [2, 17]. Given their relatively benign behavior, staging these lesions using the 7th edition guidelines creates the undesirable situation whereby a patient may be inappropriately upstaged and assigned an unnecessarily high risk. Finally, although it seems unlikely that the authors of the cSCC staging chapter intend for all other cutaneous neoplasms, including basal cell carcinomas which tend not to metastasize, as well as many tumors which behave in an essentially benign fashion, such as adnexal tumors, to receive full pathologic staging evaluation, the authors did not explicitly state this. We feel that for clarification, the authors should make some formal acknowledgment that many other cutaneous tumors do not require the full assessment needed for cSCC.

## 3. New Guidelines for Primary Tumor (T) Stage Assessment

The primary tumor (T) designation of the TNM staging system has been significantly revised in the 7th edition (Table 2). While the 2 cm size cutoff still distinguishes T1 and T2 lesions, high risk features capable of upstaging a T1 lesion to T2 have been incorporated for the first time. The specific high-risk features included are depth of invasion (>2 mm, ≥Clark level IV), perineural invasion, tumor grade (poorly differentiated or undifferentiated), and presence on high-risk anatomic sites (the lip and ear). In addition, the previous advanced T stage requirements (>5 cm for T3; presence of extradermal invasion for T4) have been replaced by involvement of specific anatomic locations (the maxilla, mandible, orbit, and temporal bone for T3 lesions, and axial or appendicular skeletal involvement or perineural involvement of the skull base for T4 lesions).

## 4. Addition of High-Risk Factors as Modifiers of T Stage

### 4.1. Histologic Differentiation

One of the high-risk features included in the 7th edition of the AJCC staging manual is the presence of poorly differentiated or undifferentiated histology. This is based on several studies which have demonstrated the relationship between high-grade (Broders class 3 and 4) and aggressive clinical behavior [5, 18]. Compared to well-differentiated tumors, poorly differentiated neoplasms have been shown to exhibit twice the local recurrence rate and three times the metastatic rate [5]. Additionally, Rowe et al. demonstrated a significant relationship between mortality and tumor grade [5], as the five-year cure rate after treatment was 61.5% for poorly differentiated cSCC, compared to 94.6% for patients with well-differentiated cSCC.

Although we feel that incorporation of tumor grade in the staging guidelines represents a significant improvement over earlier editions, we find the current description problematic, as it lacks significant details and omits several histologic features associated with high risk-disease. According to the AJCC, “high-grade tumors show poor differentiation, spindle cell characteristics, necrosis, high mitotic activity, and deep invasion” [1]. As noted by Warner and Cockerell, the description also omits several features of high-risk cSCC variants. Specifically, there is no mention of acantholytic SCC, SCC with basaloid features, SCC with a sclerosing pattern, “carcinosarcoma,” or basosquamous morphology [2]. Also absent from this description is the presence of lymphovascular invasion or inflammatory response. Although less well established than other features, there is evidence that lymphatic or vascular invasion is strongly associated with metastatic disease [18, 19]. Similarly, the presence of an inflammatory response typified by eosinophils and plasma cells has been shown to be predictive of metastases [18]. Although high mitotic activity is mentioned as a characteristic of high-grade neoplasms, there is no description of the number of mitoses required to qualify as such, nor is there any description of atypical mitoses [2]. Moreover, there is no description regarding what percentage of a neoplasm must display these features in order to be defined as “poorly differentiated” or “undifferentiated” [2]. Overall, we find the current description of histologic features of high-grade lesions significantly lacking, which limits its application in clinical practice [2].

### 4.2. Depth of Invasion/Perineural Invasion

Based on current research, depth of invasion may be the most important tumor variable associated with prognosis [18–23]. In a study by Rowe et al. [5], the presence of either tumor thickness >4 mm or depth of invasion ≥Clark level IV was associated with an increased rate of recurrence by a factor of 2, as well as an increase in the rate of metastasis by a factor of 5. Similar to melanoma, there is evidence that Breslow thickness is more important for determining prognosis in cSCC than Clark's level, although both are predictive of advanced disease [22]. Additionally, while early studies identified Breslow thickness >4 mm as predictive of aggressive behavior [5], subsequent studies have shown that a 2 mm cutoff more appropriately stratifies low and high risk lesions [18]. 

Perineural invasion has also been found to be a significant indicator of high-risk disease [5, 24–28]. Although less commonly identified (only present in 5 percent of cSCCs) [26, 27], there is some evidence that perineural invasion is associated with an increase in both the recurrence rate and the metastatic rate by a factor of 5 [29]. As perineural spread can be difficult to follow histologically and clinically, tumors can spread to great extent prior to detection [26, 27]. In general, the identification of perineural invasion carries a dismal prognosis, as the 5-year mortality rate approaches 90% [29].

Given the wealth of epidemiologic data, the authors of the 7th edition chose to include advanced tumor depth/invasion (defined as >2 mm in thickness, ≥Clark level IV) among the high-risk features capable of upstaging a T1 neoplasm. Overall, this represents an improvement over previous editions, as it allows for the identification of a subset of lesions that, although small in size, nevertheless are likely to demonstrate aggressive clinical behavior. 

However, the incorporation of these factors may be impractical for widespread use in clinical practice. As the manual does not include any exceptions to the updated staging criteria (aside from the anatomic location on vermillion or mucosal lip, eyelid, penis, or vulva, as cSCC on these sites are staged with the chapters for these anatomic locations), it implies that this data should be recorded for each and every cSCC. In particular, the accurate assessment of Breslow-type thickness requires the use of an optical micrometer and represents a significant investment of physician time and labor [2]. While in some cases performing such a thorough assessment is beneficial, for small or low-risk lesions, such thorough histopathologic staging could be viewed as a waste of valuable physician resources [2]. This is compounded by the large number of cases of cSCC, estimated to be greater than 700,000 per year in the US [30], with most of these being low-risk lesions [29]. In our opinion, very small cutaneous SCCs removed completely with initial biopsy from low-risk anatomic sites do not warrant full clinicopathologic staging evaluation and should be excluded from the new staging requirements [2]. Similarly, for verrucous variants of SCC, which may exhibit locally aggressive behavior but rarely metastasizes, full evaluation is unnecessary, and as such, it too should be included as an exception [2].

In situations where histopathologic staging is warranted, there are additional concerns regarding evaluation of tumor depth. In particular, for many initial biopsies, accurate assessment of depth is impossible, as the tumor boundary extends beyond the base of the specimen [2]. Similarly, most biopsies of large tumors are taken *in parte* at the tumor edge, precluding initial assessment of the full depth of tumor invasion. If reexcision is performed by Mohs surgeons, the debulk specimen will frequently not be sent for H&E processing. In order to obtain accurate staging information, dermatologic surgeons will need to be trained to obtain accurate assessment of vertical depth of invasion of the debulk specimen. Another concern is that many biopsy methods do not adequately maintain tissue architecture, such as shave or curettage techniques [2]. In these cases, accurate assessment of depth of invasion is not possible, even though in some of these instances, the tumor may have been already completely removed with the initial procedure.

### 4.3. High-Risk Anatomic Sites

The final high-risk feature identified in the 7th edition guidelines is the location of the primary tumor on high-risk anatomic sites. This is based on evidence that lesions located on the lip and ear are more aggressive compared to tumors present on other locations throughout the body. Based on epidemiologic data, these anatomic sites are associated with recurrence and metastatic rates between 10 to 25 percent [5, 31]. Similar to the previously mentioned high-risk variants, recognition of the increased risk associated with these sites improves the prognostic information provided by the 7th edition guidelines.

Despite this, there are specific problems worth noting regarding 7th edition's current description of high-risk anatomic sites. As described by Warner and Cockerell [2], the specific anatomic descriptions of the lip as a high-risk site are confusing and seemingly contradictory. For clarification, we are accustomed to the lip being divided into three separate anatomic regions: (1) cutaneous (hair-bearing or glabrous) lip; (2) vermillion lip (the sun exposed portion of the lip extending from the vermillion border to the line created by the opposed lips when the mouth is closed), and (3) mucosal lip (commonly referred to as the wet portion of the lip, it extends from the vermillion border posteriorly into the oral cavity) [32, 33]. Within the cutaneous carcinoma chapter of the AJCC manual, “hair-bearing lip” (an apparent reference to cutaneous lip) is defined as a high-risk anatomic site capable of upstaging a T1 neoplasm. However, a summary table within the same chapter (on page 307) refers to “non-hair-bearing lip” as the site associated with increased risk [1], a description that would seem to indicate vermillion lip. Complicating the matter further is the anatomic description of mucosal lip within the chapter on cancers of the oral cavity, which is defined as “… begin[ning] at the junction of the vermillion border with the skin and includes only the vermillion surface or that portion of the lip that comes into contact with the opposing lip” [1]. This description would seem to indicate that the region traditionally ascribed as vermillion lip should be staged along with cancers of the oral cavity, as was the case with the 6th edition [3]. Part of the source of confusion is that the vermillion outer lip and the mucosal inner lip are both lined by squamous mucosa, although the former is dry and the latter is wet mucosa. Given these confusing and seemingly contradictory statements, greater clarity is needed prior to widespread clinical adoption of these guidelines [2]. 

If tumors arising on the vermillion lip are to be staged with cancers of the oral cavity, as they were in the 6th edition [3], we find this grouping problematic [2]. SCC on the sun exposed portions of the lip, similar to cSCC, is most commonly associated with sun exposure, with total cumulative sun exposure being the most significant risk factor [31]. However, SCC originating in the oral cavity is primarily associated with alcohol or tobacco use, chronic inflammation, or HPV infection [34, 35]. As argued by Warner and Cockerell, due to the differences in etiology, these cancers may exhibit significantly different clinical behavior, making their common grouping inappropriate [2]. 

 Another limitation of the high-risk anatomic site designation is the omission of several locations associated with increased risk of recurrence or metastasis. While many high-risk areas are accounted for under separate staging guidelines (vermillion and mucosal lip, eyelid, penis, and vulva), a number of additional sites are currently unaccounted for. In particular, the central face, periorbital nose, chin, pre- and postauricular sulci, temple, mandible, scalp, temple, and dorsal hands and feet have all been associated with increased risk [27, 28, 36]. By failing to recognize the risk associated with these sites, the current guidelines may understage a subset of lesions with a propensity for more aggressive clinical behavior. We do recognize that being overly inclusive with regards to high-risk sites could be problematic, as the associated risk is not uniform across all locations. However, we agree with Warner and Cockerell that the central face and dorsal hands and feet warrant strong consideration for subsequent editions [2].

### 4.4. Pertinent High-Risk Factors Omitted in the 7th Edition

While the inclusion of selected high-risk features represents a significant improvement over previous editions, several other variables associated with aggressive tumor behavior were omitted and bear mention, including the presence of immunosupression, occurrence of cSCC at sites of prior chronic inflammation/burn/scar, and recurrence of previously treated tumors. 

One of the most important risk factors associated with cSCC is host immune status. Numerous studies have displayed the significant impact immunosuppression has on both the incidence and biologic characteristics of cSCC [5, 37, 38]. cSCC tumors in immunosuppressed patients exhibit a strong propensity to recur and metastasize and frequently exhibit aggressive behavior irrespective of size [37, 38]. While the authors of the 7th edition acknowledge the impact immune status has on prognosis, they chose not to include it, as TNM staging is focused primarily on tumor characteristics rather than host factors. Rather, they recommend institutions wishing to record immune status data include an “I” qualifier in the final stage to denote immunosuppression [1]. Although we understand the authors' concerns regarding the inclusion of patient characteristics into a staging protocol focused on tumor-specific variables, we feel that they should have taken a more aggressive stance regarding documentation of immunosuppression. We feel that a system whereby immune status data is recorded in all cases, using an “IS” designation for immunosuppression and “IC” for immunocompetent would dramatically improve the epidemiologic data available by helping elucidate the role immune status plays regarding clinical behavior and prognosis [2]. Moreover, by not including this high-risk variable, the current system misses a subset of patients who, despite having tumors small in size, are likely to experience an aggressive clinical course [38].

cSCC that arise within scars, sinus tracts, burns, or in the setting of a chronic inflammation have been shown to exhibit more aggressive clinical behavior and a greater propensity to metastasize [5, 39, 40]. The short-term metastatic rate for these lesions is roughly 25 percent, while the overall metastatic rate approaches 40 percent [5]. Although these lesions are very rare, representing less than 1% of metastatic lesions [5], given the significant risk associated with them, we agree with Warner and Cockerell that the AJCC should give strong consideration to including these lesions among the high-risk factors capable of upstaging a small neoplasm [2]. 

Recurrent disease is an additional factor worth noting. The presence of recurrent or persistent disease is a strong prognostic variable for metastasis and control of local disease [5, 41]. Recurrent or previously treated tumors tend to be more aggressive, less responsive to treatment, and associated with decreased survival (78% 5-year survival compared to 97% for primary lesions) [42]. Although rarely performed for cutaneous SCC, under the current TNM staging system, recurrent neoplasms are denoted with an “r” qualifier prior to TNM-specific designations (for example, rT2N0M0 for a locally recurrent tumor greater than 2 cm in size, without evidence of lymph node involvement or metastasis) [1, 39]. As a result, a subset of high-risk lesions are clearly defined as such, and data collection for these tumors is significantly enhanced. However, in light of most recent changes to T staging criteria, a recurrent neoplasm less than 2 cm with only 1 of the three high-risk features would be classified as a Stage I neoplasm rather than Stage II, despite its less favorable prognosis. Given this, we feel that recurrent or previously treated neoplasms should be included among the high-risk features capable of upstaging a neoplasm. Importantly, we do not recommend altering the current “r” qualifier, as this designation helps facilitate improved epidemiologic data.

## 5. Changes in Advanced T Stage Designation

In the 7th edition staging manual, T3 tumors are now classified as those with bony extension to the mandible, maxilla, temple, or orbit whereas the T4 designation is reserved for perineural involvement of the skull base or bony extension to the axial or appendicular skeleton. More practical than previous versions, these specific designations represent an attempt to achieve greater congruence with head and neck cancer guidelines [3].

A drawback to the new guidelines pertaining to advanced T stage is the inclusion of appendicular skeletal involvement with T4 lesions. It seems inappropriate to assign the same prognosis to an SCC invading the small bones of the hand or foot with one displaying perineural involvement of the skull base [2, 39]. Similarly, under this system, a neoplasm with bony extension into the hand or foot would be assigned a higher classification than one invading the maxilla, mandible, temple, or orbit, a distinction that seems largely inappropriate. In addition, as T4 neoplasms are automatically classified as Stage IV, there is the potential for patients to be given a significantly worse prognosis, and subsequently a more aggressive treatment regimen, than their primary cancer may warrant. As advocated by Warner and Cockerell, we feel that appendicular bone involvement should be grouped with T3 lesions rather than T4 [2].

Although not a significant issue, a potential drawback of the new T stages as they currently stand is that only cSCC arising on the head or neck is capable of receiving T3 classification. While this likely represents an attempt to achieve greater congruence with the head and neck staging protocol, it seems awkwardly restrictive, particularly for guidelines pertaining to the entire external body surface.

## 6. Changes in Stratification of Lymph Node Status

The nodal (N) staging system has been completely revised compared to previous editions. In the past, only the presence or absence of nodal metastasis was recorded, leaving no way to differentiate a patient with sentinel node involvement versus a patient with bilateral multinodal disease [5, 39]. In the new guidelines, node size, number of involved nodes, and the presence of contralateral or bilateral node involvement are all included to better stratify patients. The inclusion of these variables is the result of significant evidence that prognosis decreases with advancing nodal burden  [42]. Under the new guidelines (Table 3), metastasis to a single node less than 3 cm in greatest dimension is defined as N1. The N2 designation refers to either a single node 3–6 cm in size, or multinodal disease where no individual node is greater than 6 cm in size. Based on the specific pattern of nodal involvement, N2 is subcategorized into three separate groupings. Involvement of a single ipsilateral node is categorized as N2a, metastasis to multiple ipsilateral nodes as N2b, and involvement of contralateral or bilateral lymph nodes as N2c. The N3 designation is reserved for any lymph node greater than 6 cm in greatest dimension, regardless of number of nodes involved.

In general, the recommendations regarding the nodal staging are largely beneficial, with only minor issues worth noting. Under the new staging criteria, there is no mechanism for recognizing ipsilateral progressive nodal disease beyond the single and multinodal distinctions. Additionally, although it is likely that spread to distant lymph node basins is associated with a worse prognosis than regional nodal involvement, there is no mechanism for assigning this risk unless there is spread to the contralateral side of the body. Including these factors within staging guidelines would lead to greater patient stratification and improve the accuracy of tumor-specific prognosis, although we recognize that such a system designed to apply to the whole body may be cumbersome and difficult to apply to clinical practice. 

Two additional omissions from the N staging criteria are extracapsular invasion and micrometastasis. The absence of micrometastasis is not surprising as this feature has only recently gained recognition in staging guidelines for other anatomic sites and is not frequently associated with SCC. The omission of extracapsular involvement is more curious as there is a precedent for its inclusion in other chapters. In particular the guidelines for the penis, vulva, and head and neck all contain some provision for recognizing extracapsular invasion [1]. Finally, similar to our recommendations regarding T staging, we feel that full nodal assessment is largely unnecessary for small superficial cSCC cured with initial biopsy and verrucous cSCC (as well as being unnecessary for basal cell carcinomas and cutaneous tumors that behave in a benign fashion) [2].

## 7. M Staging Guidelines

No changes were made to the distant metastasis designation of the AJCC cutaneous SCC staging system (Table 4). Importantly, there is no provision for classification of lymph node metastasis far removed from regional nodal basins as distant metastasis. This is in contrast to head and neck carcinomas, for which the AJCC defines mediastinal lymph node involvement excluding level VII as distant metastases [1]. With this in mind, it might have been better to classify metastases to mediastinal and retroperitoneal lymph nodes as M1 lesions [2], although we recognize that it may be problematic to define specific nodal locations as distant metastases for a staging protocol applied to the entire skin surface.

## 8. Anatomic Stage/Prognostic Groups Designation

Given the changes to the T and N classifications, the staging groups offer reasonable stratification of patients based on prognosis (Table 5). Briefly, T1 and T2 tumors are assigned Stage I and Stage II, respectively, Stage III includes all T3 or N1 tumors that do not meet criteria for Stage IV, and the presence of any T4, N2-3, or M1 designation is required for Stage IV classification.

One potential oversight is that the improved patient stratification afforded by the new N guidelines is largely lost when applied to the staging group, as any neoplasm receiving an N2 or N3 designation is automatically assigned Stage IV. In order to more clearly distinguish N2 and N3 lesions, it has been suggested that separate IVa and IVb groupings could be created [2]. However, we recognize that this may not be a feasible solution, as T4 or M1 lesions are also classified as Stage IV, irrespective of N status. As mentioned previously, the inclusion of T4 tumors with the Stage IV grouping raises further concerns regarding the current inclusion of invasion of the appendicular skeleton within T4 criteria.

## 9. Conclusions

Designing a comprehensive set of staging guidelines that are accurate, easily applied, and efficient represents a tremendous challenge, one to which there is likely no perfect solution. To that end, we applaud the authors of the seventh edition for creating a set of guidelines that, although imperfect, represent a significant improvement over previous editions. In particular, the creation of guidelines oriented specifically towards cutaneous squamous cell carcinoma enhances the ability to accurately assess the prognosis associated with these types of lesions. Furthermore, incorporation of high risk features into the new cSCC guidelines helps better stratify patients and assess tumor-specific risk. However, despite these efforts, the guidelines still contain a number of significant limitations. In particular, several recommendations are largely impractical. Specifically, we feel that it is neither economically feasible nor medically justified to perform full clinicopathologic staging criteria on the verrucous variant of cSCC and small superficial SCC cured with primary biopsy. Similarly, for cutaneous neoplasms that rarely metastasize, such as basal cell carcinoma, full staging is unnecessary and should be mentioned in the staging guidelines as an exception. In other cases, although relevant, full initial evaluation of high-risk features may not be possible, as primary biopsies frequently involve only a small portion of the primary tumor, precluding accurate assessment. Moreover, Mohs surgeons will need to alter their clinical practice and either routinely send a debulking specimen for H&E processing, or begin assessing for vertical depth of invasion. Other issues that need to be addressed include the lack of clear or comprehensive definitions pertaining to the anatomy of the lip and the histologic designation of “poorly differentiated” and “undifferentiated” neoplasms. Moreover, future additions should include important high-risk factors including SCC arising at sites of burns/scars/sinus tracts/chronic inflammation and/or in certain other high-risk anatomic sites not currently included in the staging criteria, and the presence of recurrent or previously treated disease. Additionally, the current classification of cSCC with appendicular involvement with T4 lesions is largely inappropriate, and these lesions would be more appropriately designated as T3 lesions. Addressing these concerns in subsequent editions would go a long way toward improving the accuracy, applicability, and practicality of these guidelines.

## Figures and Tables

**Table 1:** AJCC cancer staging manual summary of changes from the sixth edition to the seventh edition.

(i) The chapter entitled “Carcinoma of the Skin,” has been eliminated and two chapters have been created in its place:
(ii) “Merkel Cell Carcinoma”: an entirely new chapter specifically for Merkel cell carcinoma (MCC) has been designed (see Chapter 30).
(iii) This chapter has been renamed “Cutaneous Squamous Cell Carcinoma and Other Cutaneous Carcinomas” and is an entirely new staging system that, for the first time, reflects a multidisciplinary effort to provide a mechanism for staging nonmelanoma skin cancers according to evidence-based medicine. The title of this chapter reflects the basis of the data, which is focused on cutaneous squamous cell carcinoma (cSCC). All other nonmelanoma skin carcinomas (except Merkel cell carcinoma) are also to be staged according to the cSCC system.
(iv) Anatomic site of the eyelid is excluded—cSCC of the eyelid are staged by ophthalmic carcinoma of the eyelid (see Chapter 48). Cutaneous SCC of the penis, vulva, and mucosal lip are also excluded, as they are staged with chapters specific for those anatomic sites.
(v) The T staging has eliminated the five-centimeter-size breakpoint and invasion of extradermal structures for T4. Two centimeters continues to differentiate T1 and 2; however, a list of clinical and histologic “high-risk features” have been created that can increase the T staging, independent of tumor size.
(vi) Grade has been included as one of the “high-risk features” within the T category and now contributes toward the final stage grouping. Other “high-risk features” include primary anatomic site of ear or hair-bearing lip, invasion greater than two millimeters depth, Clark's level greater than or equal to IV, or perineural invasion.
(vii) Advanced T stage is reserved for invasion of specific anatomic sites (maxilla, mandible, orbit, or temporal bone involvement for T3; appendicular or axial skeletal involvement or perineural invasion of the skull base for T4).
(viii) Nodal (N) staging has been completely revised to reflect published evidence-based data demonstrating that survival decreases with increasing nodal size and number of nodes involved.
(ix) Because the majority of cSCC tumors occur on the head and neck, the seventh edition staging system for cSCC and other cutaneous carcinomas was made congruent with the AJCC head and neck staging system.

From the AJCC 7th edition “Cutaneous Squamous Cell Carcinoma and Other Cutaneous Carcinomas”.

**Table 2:** Primary tumor (T)*.

Tx	Primary tumor cannot be assessed
T0	No evidence of primary tumor
Tis	Carcinoma in situ
T1	Carcinoma less than 2 cm in greatest dimension, with less than 2 high risk features**
T2	Carcinoma greater than 2 cm in greatest dimension, Or Tumor of any size with at least 2 high risk features**
T3	Tumor invasion of the maxilla, mandible, orbit, or temporal bone
T4	Tumor invasion of the skeleton (appendicular or axial) or with perineural involvement of the skull base

*Excludes cutaneous SCC of the eyelid.

**High-risk features for the primary tumor (T) staging:

Depth/invasion: >2 mm thickness, Clark level ≥IV, Perineural invasion.

Anatomic location: Primary site ear, Primary site non-hair-bearing lip.

Differentiation: Poorly differentiated or undifferentiated.

From the AJCC 7th edition “Cutaneous Squamous Cell Carcinoma and Other Cutaneous Carcinomas”.

**Table 3:** Regional lymph nodes (N).

Nx	Regional lymph nodes cannot be assessed.
N0	No regional lymph node metastases.
N1	Metastasis in a single ipsilateral lymph node, 3 cm or less in greatest dimension.
N2	Metastasis in a single ipsilateral lymph node, more than 3 cm but not more than 6 cm in greatest dimension; or in multiple ipsilateral lymphnodes, none more than 6 cm in greatest dimension; or in bilateral orcontralateral lymph nodes, none more than 6 cm in greatest dimension.
N2a	Metastasis in a single ipsilateral lymph node, more than 3 cm but not more than 6 cm in greatest dimension.
N2b	Metastasis in multiple ipsilateral lymph nodes, none more than 6 cm ingreatest dimension.
N2c	Metastasis in bilateral or contralateral lymph nodes, none more than 6 cm in greatest dimension.
N3	Metastasis in a lymph node, more than 6 cm in greatest dimension.

From the AJCC 7th edition “Cutaneous Squamous Cell Carcinoma and Other Cutaneous Carcinomas”.

**Table 4:** Distant metastasis (M).

M0	No distant metastasis
M1	Distant metastasis

From the AJCC 7th edition “Cutaneous Squamous Cell Carcinoma and Other Cutaneous Carcinomas”.

**Table 5:** Anatomic stage/prognostic groups.

Stage 0	Tis	N0	M0
Stage I	T1	N0	M0
Stage II	T2	N0	M0
Stage III	T3	N0	M0
	T1	N1	M0
	T2	N1	M0
	T3	N1	M0
Stage IV	T1	N2	M0
	T2	N2	M0
	T3	N2	M0
	T Any	N3	M0
	T4	N Any	M0
	T Any	N Any	M1

From the AJCC 7th edition “Cutaneous Squamous Cell Carcinoma and Other Cutaneous Carcinomas”.
